# Impact of electrolyte imbalances on the outcome of aneurysmal subarachnoid hemorrhage: a prospective study

**Published:** 2012-11

**Authors:** Mohammad Shirani, Seyed Maysam Alimohamadi

**Affiliations:** ^a^Neuroendocrine Surgery Center, Sina Hospital, Tehran University of medical Sciences, Tehran, Iran.

## Abstract

**Background:**

Electrolyte disturbances are frequently observed during the acute and subacute period after subarachnoid hemorrhage (SAH) that may potentially worsen the therapeutic outcomes. This study was aimed to determine the pattern of electrolyte disturbance in the acute and subacute phase after SAH and their potential impacts on the long term outcome of the patients.

**Methods:**

A total of 53 patients were prospectively enrolled in the study. A standard and uniform medical care was performed for all patients. The serum levels of electrolytes (Sodium, Potassium and Magnesium) were measured on admission, 3-5, and 7-10 days after SAH. Radiographic intensity of hemorrhage (Fisher’s scale), and the clinical grading (WFNS grade) were documented in the first visit. The outcomes were evaluated using Glasgow outcome scale (GOS) at 3 months after discharge.

**Results:**

Hyponatremia was the most common electrolyte imbalance among the patients but did not worsen the outcome. Although less common, hypernatremia in the subacute phase was significantly associated with poor outcome. Both hypokalemia and hypomagnesemia were predictive of poor outcomes.

**Conclusions:**

Because electrolyte abnormalities can adversely affect the outcome, the serum levels of electrolytes should be closely monitored with serial measurements and treated properly in patients with aneurysmal SAH.

**Keywords:**

Subarachnoid hemorrhage, Aneurysm, Electrolyte imbalance, Outcome


					Volume 4, Suppl. 1 Nov 2012
Publisher: Kermanshah University of Medical Sciences
URL: http://www.jivresearch.org
Copyright:
Congress of Iranian Neurosurgeons, 14-16 November 2012, Kermanshah, Iran
Paper No. 28
Impact of electrolyte imbalances on the outcome of
aneurysmal subarachnoid hemorrhage: a prospective study
Mohammad Shirani a
, Seyed Maysam Alimohamadi a,*
a
Neuroendocrine Surgery Center, Sina Hospital, Tehran University of medical Sciences, Tehran, Iran.
Abstract:
Background: Electrolyte disturbances are frequently observed during the acute and subacute period after
subarachnoid hemorrhage (SAH) that may potentially worsen the therapeutic outcomes. This study was aimed to
determine the pattern of electrolyte disturbance in the acute and subacute phase after SAH and their potential
impacts on the long term outcome of the patients.
Methods: A total of 53 patients were prospectively enrolled in the study. A standard and uniform medical care was
performed for all patients. The serum levels of electrolytes (Sodium, Potassium and Magnesium) were measured on
admission, 3-5, and 7-10 days after SAH. Radiographic intensity of hemorrhage (Fisher's scale), and the clinical
grading (WFNS grade) were documented in the first visit. The outcomes were evaluated using Glasgow outcome
scale (GOS) at 3 months after discharge.
Results: Hyponatremia was the most common electrolyte imbalance among the patients but did not worsen the
outcome. Although less common, hypernatremia in the subacute phase was significantly associated with poor outcome.
Both hypokalemia and hypomagnesemia were predictive of poor outcomes.
Conclusions: Because electrolyte abnormalities can adversely affect the outcome, the serum levels of electrolytes
should be closely monitored with serial measurements and treated properly in patients with aneurysmal SAH.
Keywords:
Subarachnoid hemorrhage, Aneurysm, Electrolyte imbalance, Outcome
* Corresponding Author at:
Seyed Maysam Alimohamadi: Tehran University of medical Sciences, Sina Hospital Neuroendocrine Surgery Center, Tehran, Iran. Tel: 09123270725,
Email: alimohamadi59@gmail.com, (Alimohamadi SM.).

				

